# Supplementary material for: Modeling glioblastoma heterogeneity as a dynamic network of cell states
Source: Mol Syst Biol. 2021 Sep 16;17(9):e10105. doi: 10.15252/msb.202010105 (PMC8444284; doi:10.15252/msb.202010105)
Supplement: Supplementary file 5 — Source Data for Figure 3 [file MSB-17-e10105-s001.zip › Figure3A_sourcedata/GSEA_3065/nosigdb_state2.GseaPreranked.1622718419215/GAROFANO_IAVARONE_PPR_SUBTYPE.html]

Details for gene set GAROFANO\_IAVARONE\_PPR\_SUBTYPE[GSEA]

|  || Dataset | state2 |
| Phenotype | NoPhenotypeAvailable |
| Upregulated in class | na\_pos |
| GeneSet | GAROFANO\_IAVARONE\_PPR\_SUBTYPE |
| Enrichment Score (ES) | 0.77930367 |
| Normalized Enrichment Score (NES) | 2.9729235 |
| Nominal p-value | 0.0 |
| FDR q-value | 0.0 |
| FWER p-Value | 0.0 |
Table: GSEA Results Summary

  

Fig 1: Enrichment plot: GAROFANO\_IAVARONE\_PPR\_SUBTYPE      
 Profile of the Running ES Score & Positions of GeneSet Members on the Rank Ordered List

  

| PROBE | GENE SYMBOL | GENE\_TITLE | RANK IN GENE LIST | RANK METRIC SCORE | RUNNING ES | CORE ENRICHMENT || 1 | HIST1H4C |  |  | 0 | 1.309 | 0.0156 | Yes |
| 2 | HIST1H1B |  |  | 1 | 0.902 | 0.0263 | Yes |
| 3 | HMGB2 |  |  | 4 | 0.823 | 0.0359 | Yes |
| 4 | TYMS |  |  | 5 | 0.792 | 0.0453 | Yes |
| 5 | H2AFZ |  |  | 8 | 0.735 | 0.0539 | Yes |
| 6 | UBE2C |  |  | 9 | 0.704 | 0.0623 | Yes |
| 7 | H2AFX |  |  | 10 | 0.675 | 0.0703 | Yes |
| 8 | UBE2T |  |  | 12 | 0.643 | 0.0778 | Yes |
| 9 | MCM7 |  |  | 13 | 0.626 | 0.0853 | Yes |
| 10 | MYBL2 |  |  | 14 | 0.600 | 0.0924 | Yes |
| 11 | TOP2A |  |  | 15 | 0.596 | 0.0995 | Yes |
| 12 | PCNA |  |  | 16 | 0.595 | 0.1066 | Yes |
| 13 | MKI67 |  |  | 17 | 0.590 | 0.1136 | Yes |
| 14 | CDK1 |  |  | 18 | 0.577 | 0.1205 | Yes |
| 15 | UBE2S |  |  | 19 | 0.574 | 0.1274 | Yes |
| 16 | MAD2L1 |  |  | 20 | 0.563 | 0.1341 | Yes |
| 17 | ATAD2 |  |  | 21 | 0.563 | 0.1408 | Yes |
| 18 | RRM2 |  |  | 22 | 0.556 | 0.1474 | Yes |
| 19 | CKS1B |  |  | 23 | 0.554 | 0.1540 | Yes |
| 20 | DUT |  |  | 24 | 0.552 | 0.1606 | Yes |
| 21 | HMGB1 |  |  | 25 | 0.549 | 0.1671 | Yes |
| 22 | NUDT1 |  |  | 26 | 0.541 | 0.1735 | Yes |
| 23 | CLSPN |  |  | 27 | 0.536 | 0.1799 | Yes |
| 24 | TPX2 |  |  | 29 | 0.531 | 0.1861 | Yes |
| 25 | ESCO2 |  |  | 30 | 0.530 | 0.1924 | Yes |
| 26 | FANCI |  |  | 32 | 0.521 | 0.1985 | Yes |
| 27 | STMN1 |  |  | 33 | 0.520 | 0.2047 | Yes |
| 28 | CENPF |  |  | 34 | 0.518 | 0.2109 | Yes |
| 29 | HMGN2 |  |  | 35 | 0.512 | 0.2170 | Yes |
| 30 | GINS2 |  |  | 37 | 0.506 | 0.2229 | Yes |
| 31 | SMC2 |  |  | 39 | 0.493 | 0.2287 | Yes |
| 32 | DBF4 |  |  | 41 | 0.492 | 0.2344 | Yes |
| 33 | SMC4 |  |  | 42 | 0.489 | 0.2403 | Yes |
| 34 | TMPO |  |  | 43 | 0.489 | 0.2461 | Yes |
| 35 | CENPK |  |  | 44 | 0.488 | 0.2519 | Yes |
| 36 | ZWINT |  |  | 45 | 0.488 | 0.2577 | Yes |
| 37 | NUSAP1 |  |  | 47 | 0.483 | 0.2633 | Yes |
| 38 | PTMA |  |  | 49 | 0.474 | 0.2689 | Yes |
| 39 | KPNA2 |  |  | 50 | 0.472 | 0.2745 | Yes |
| 40 | TUBA1B |  |  | 52 | 0.462 | 0.2799 | Yes |
| 41 | RFC2 |  |  | 53 | 0.460 | 0.2854 | Yes |
| 42 | CENPM |  |  | 54 | 0.460 | 0.2908 | Yes |
| 43 | CHAF1A |  |  | 55 | 0.457 | 0.2963 | Yes |
| 44 | TK1 |  |  | 56 | 0.456 | 0.3017 | Yes |
| 45 | NCAPG |  |  | 60 | 0.445 | 0.3067 | Yes |
| 46 | BIRC5 |  |  | 61 | 0.443 | 0.3119 | Yes |
| 47 | GMNN |  |  | 64 | 0.435 | 0.3169 | Yes |
| 48 | RANBP1 |  |  | 65 | 0.430 | 0.3220 | Yes |
| 49 | LMNB1 |  |  | 69 | 0.417 | 0.3267 | Yes |
| 50 | EZH2 |  |  | 70 | 0.417 | 0.3316 | Yes |
| 51 | CENPH |  |  | 71 | 0.412 | 0.3365 | Yes |
| 52 | RRM1 |  |  | 72 | 0.412 | 0.3414 | Yes |
| 53 | LSM4 |  |  | 73 | 0.410 | 0.3463 | Yes |
| 54 | ASF1B |  |  | 76 | 0.407 | 0.3509 | Yes |
| 55 | NUCKS1 |  |  | 78 | 0.404 | 0.3556 | Yes |
| 56 | MCM4 |  |  | 79 | 0.399 | 0.3604 | Yes |
| 57 | H2AFV |  |  | 82 | 0.397 | 0.3649 | Yes |
| 58 | MCM5 |  |  | 83 | 0.394 | 0.3696 | Yes |
| 59 | KIF4A |  |  | 84 | 0.393 | 0.3743 | Yes |
| 60 | LSM5 |  |  | 85 | 0.393 | 0.3789 | Yes |
| 61 | KIF15 |  |  | 86 | 0.392 | 0.3836 | Yes |
| 62 | CENPN |  |  | 87 | 0.390 | 0.3882 | Yes |
| 63 | HNRNPD |  |  | 88 | 0.390 | 0.3929 | Yes |
| 64 | GTSE1 |  |  | 89 | 0.389 | 0.3975 | Yes |
| 65 | RAD51AP1 |  |  | 90 | 0.388 | 0.4021 | Yes |
| 66 | BRCA1 |  |  | 92 | 0.381 | 0.4066 | Yes |
| 67 | DTYMK |  |  | 94 | 0.379 | 0.4110 | Yes |
| 68 | CDC45 |  |  | 95 | 0.377 | 0.4154 | Yes |
| 69 | MND1 |  |  | 96 | 0.376 | 0.4199 | Yes |
| 70 | MCM10 |  |  | 100 | 0.373 | 0.4240 | Yes |
| 71 | CDCA5 |  |  | 101 | 0.373 | 0.4285 | Yes |
| 72 | DNAJC9 |  |  | 103 | 0.373 | 0.4328 | Yes |
| 73 | SPC25 |  |  | 104 | 0.372 | 0.4372 | Yes |
| 74 | CENPE |  |  | 105 | 0.372 | 0.4417 | Yes |
| 75 | CDC6 |  |  | 106 | 0.372 | 0.4461 | Yes |
| 76 | FEN1 |  |  | 107 | 0.371 | 0.4505 | Yes |
| 77 | CDT1 |  |  | 108 | 0.368 | 0.4549 | Yes |
| 78 | HNRNPAB |  |  | 113 | 0.357 | 0.4587 | Yes |
| 79 | NDC80 |  |  | 115 | 0.354 | 0.4628 | Yes |
| 80 | KIF23 |  |  | 117 | 0.351 | 0.4669 | Yes |
| 81 | POP7 |  |  | 119 | 0.347 | 0.4709 | Yes |
| 82 | POLD3 |  |  | 120 | 0.347 | 0.4750 | Yes |
| 83 | DNMT1 |  |  | 121 | 0.345 | 0.4791 | Yes |
| 84 | ASPM |  |  | 122 | 0.342 | 0.4832 | Yes |
| 85 | KIF11 |  |  | 129 | 0.335 | 0.4865 | Yes |
| 86 | TACC3 |  |  | 130 | 0.334 | 0.4905 | Yes |
| 87 | MELK |  |  | 133 | 0.332 | 0.4942 | Yes |
| 88 | POC1A |  |  | 134 | 0.332 | 0.4982 | Yes |
| 89 | CCNA2 |  |  | 135 | 0.331 | 0.5021 | Yes |
| 90 | ANP32E |  |  | 136 | 0.330 | 0.5061 | Yes |
| 91 | ARHGAP11A |  |  | 145 | 0.321 | 0.5090 | Yes |
| 92 | NCL |  |  | 146 | 0.316 | 0.5128 | Yes |
| 93 | DTL |  |  | 148 | 0.311 | 0.5164 | Yes |
| 94 | MCM6 |  |  | 149 | 0.311 | 0.5201 | Yes |
| 95 | CDCA2 |  |  | 150 | 0.309 | 0.5237 | Yes |
| 96 | CKAP2 |  |  | 151 | 0.308 | 0.5274 | Yes |
| 97 | CBX3 |  |  | 152 | 0.308 | 0.5311 | Yes |
| 98 | KIF22 |  |  | 154 | 0.306 | 0.5346 | Yes |
| 99 | NASP |  |  | 156 | 0.306 | 0.5381 | Yes |
| 100 | HELLS |  |  | 161 | 0.304 | 0.5413 | Yes |
| 101 | AURKB |  |  | 163 | 0.303 | 0.5448 | Yes |
| 102 | SAP30 |  |  | 164 | 0.303 | 0.5484 | Yes |
| 103 | LMNB2 |  |  | 169 | 0.298 | 0.5515 | Yes |
| 104 | ECT2 |  |  | 171 | 0.297 | 0.5549 | Yes |
| 105 | DCTPP1 |  |  | 172 | 0.296 | 0.5585 | Yes |
| 106 | BARD1 |  |  | 185 | 0.292 | 0.5606 | Yes |
| 107 | NUF2 |  |  | 188 | 0.287 | 0.5638 | Yes |
| 108 | PPIA |  |  | 189 | 0.287 | 0.5672 | Yes |
| 109 | CCNB2 |  |  | 190 | 0.286 | 0.5706 | Yes |
| 110 | FAM111B |  |  | 191 | 0.285 | 0.5740 | Yes |
| 111 | HAUS1 |  |  | 192 | 0.285 | 0.5774 | Yes |
| 112 | PARP1 |  |  | 194 | 0.284 | 0.5807 | Yes |
| 113 | SMC3 |  |  | 197 | 0.283 | 0.5838 | Yes |
| 114 | HMGB3 |  |  | 200 | 0.282 | 0.5870 | Yes |
| 115 | NCAPG2 |  |  | 201 | 0.282 | 0.5903 | Yes |
| 116 | FOXM1 |  |  | 202 | 0.282 | 0.5937 | Yes |
| 117 | PAFAH1B3 |  |  | 203 | 0.282 | 0.5971 | Yes |
| 118 | HJURP |  |  | 209 | 0.277 | 0.5998 | Yes |
| 119 | DSN1 |  |  | 216 | 0.273 | 0.6024 | Yes |
| 120 | ATAD5 |  |  | 222 | 0.269 | 0.6050 | Yes |
| 121 | CHEK1 |  |  | 224 | 0.266 | 0.6081 | Yes |
| 122 | CBX5 |  |  | 225 | 0.265 | 0.6112 | Yes |
| 123 | MCM2 |  |  | 228 | 0.262 | 0.6141 | Yes |
| 124 | CENPA |  |  | 238 | 0.259 | 0.6162 | Yes |
| 125 | SMC1A |  |  | 249 | 0.253 | 0.6181 | Yes |
| 126 | KIF2C |  |  | 252 | 0.251 | 0.6209 | Yes |
| 127 | CKLF |  |  | 254 | 0.249 | 0.6237 | Yes |
| 128 | SPC24 |  |  | 256 | 0.248 | 0.6266 | Yes |
| 129 | LIG1 |  |  | 259 | 0.246 | 0.6293 | Yes |
| 130 | MSH2 |  |  | 266 | 0.244 | 0.6315 | Yes |
| 131 | HIST2H2AC |  |  | 267 | 0.244 | 0.6344 | Yes |
| 132 | HNRNPR |  |  | 269 | 0.244 | 0.6372 | Yes |
| 133 | TIMELESS |  |  | 272 | 0.243 | 0.6399 | Yes |
| 134 | SAE1 |  |  | 278 | 0.242 | 0.6422 | Yes |
| 135 | HAUS8 |  |  | 280 | 0.242 | 0.6450 | Yes |
| 136 | CEP78 |  |  | 283 | 0.239 | 0.6476 | Yes |
| 137 | TRIP13 |  |  | 286 | 0.237 | 0.6502 | Yes |
| 138 | MAZ |  |  | 290 | 0.236 | 0.6527 | Yes |
| 139 | NCAPD3 |  |  | 294 | 0.234 | 0.6552 | Yes |
| 140 | EXOSC9 |  |  | 298 | 0.233 | 0.6576 | Yes |
| 141 | HNRNPA0 |  |  | 299 | 0.232 | 0.6604 | Yes |
| 142 | KIF14 |  |  | 302 | 0.231 | 0.6629 | Yes |
| 143 | BAZ1B |  |  | 303 | 0.230 | 0.6656 | Yes |
| 144 | SSRP1 |  |  | 319 | 0.226 | 0.6667 | Yes |
| 145 | CKAP2L |  |  | 327 | 0.224 | 0.6686 | Yes |
| 146 | TMEM97 |  |  | 331 | 0.223 | 0.6709 | Yes |
| 147 | RACGAP1 |  |  | 333 | 0.222 | 0.6734 | Yes |
| 148 | TRIM28 |  |  | 334 | 0.222 | 0.6761 | Yes |
| 149 | BUB1 |  |  | 335 | 0.222 | 0.6787 | Yes |
| 150 | NCAPH |  |  | 336 | 0.222 | 0.6813 | Yes |
| 151 | PKMYT1 |  |  | 342 | 0.220 | 0.6834 | Yes |
| 152 | HIRIP3 |  |  | 347 | 0.217 | 0.6855 | Yes |
| 153 | BLM |  |  | 355 | 0.215 | 0.6873 | Yes |
| 154 | MASTL |  |  | 357 | 0.215 | 0.6898 | Yes |
| 155 | PLK1 |  |  | 364 | 0.213 | 0.6916 | Yes |
| 156 | POLA2 |  |  | 366 | 0.212 | 0.6941 | Yes |
| 157 | RMI1 |  |  | 372 | 0.211 | 0.6960 | Yes |
| 158 | CENPW |  |  | 373 | 0.211 | 0.6985 | Yes |
| 159 | ZWILCH |  |  | 374 | 0.210 | 0.7010 | Yes |
| 160 | MSH6 |  |  | 380 | 0.209 | 0.7030 | Yes |
| 161 | CDK2 |  |  | 382 | 0.208 | 0.7053 | Yes |
| 162 | DSCC1 |  |  | 395 | 0.202 | 0.7064 | Yes |
| 163 | HNRNPM |  |  | 412 | 0.198 | 0.7070 | Yes |
| 164 | DPF1 |  |  | 416 | 0.197 | 0.7090 | Yes |
| 165 | KIF18A |  |  | 419 | 0.195 | 0.7111 | Yes |
| 166 | SKA2 |  |  | 421 | 0.195 | 0.7133 | Yes |
| 167 | BRIP1 |  |  | 426 | 0.194 | 0.7152 | Yes |
| 168 | BRCA2 |  |  | 427 | 0.193 | 0.7175 | Yes |
| 169 | PXMP2 |  |  | 430 | 0.193 | 0.7195 | Yes |
| 170 | TROAP |  |  | 435 | 0.193 | 0.7214 | Yes |
| 171 | SPAG5 |  |  | 439 | 0.192 | 0.7233 | Yes |
| 172 | FANCD2 |  |  | 447 | 0.191 | 0.7248 | Yes |
| 173 | XRCC2 |  |  | 455 | 0.190 | 0.7263 | Yes |
| 174 | EXOSC8 |  |  | 456 | 0.189 | 0.7286 | Yes |
| 175 | MAD2L2 |  |  | 460 | 0.189 | 0.7305 | Yes |
| 176 | RTKN2 |  |  | 463 | 0.188 | 0.7325 | Yes |
| 177 | GINS1 |  |  | 470 | 0.185 | 0.7340 | Yes |
| 178 | BRD7 |  |  | 477 | 0.184 | 0.7356 | Yes |
| 179 | SKA1 |  |  | 497 | 0.182 | 0.7356 | Yes |
| 180 | DEPDC1B |  |  | 505 | 0.179 | 0.7370 | Yes |
| 181 | HNRNPA2B1 |  |  | 510 | 0.178 | 0.7386 | Yes |
| 182 | PRC1 |  |  | 514 | 0.178 | 0.7404 | Yes |
| 183 | KHDRBS1 |  |  | 548 | 0.173 | 0.7388 | Yes |
| 184 | RIF1 |  |  | 552 | 0.172 | 0.7405 | Yes |
| 185 | CENPP |  |  | 558 | 0.171 | 0.7420 | Yes |
| 186 | BUB1B |  |  | 572 | 0.168 | 0.7426 | Yes |
| 187 | KIFC1 |  |  | 577 | 0.167 | 0.7441 | Yes |
| 188 | CDC25A |  |  | 578 | 0.167 | 0.7461 | Yes |
| 189 | CKAP5 |  |  | 596 | 0.164 | 0.7462 | Yes |
| 190 | CASP8AP2 |  |  | 608 | 0.162 | 0.7469 | Yes |
| 191 | CENPO |  |  | 609 | 0.162 | 0.7488 | Yes |
| 192 | RFC3 |  |  | 617 | 0.161 | 0.7499 | Yes |
| 193 | ING1 |  |  | 631 | 0.159 | 0.7504 | Yes |
| 194 | CDCA8 |  |  | 634 | 0.158 | 0.7520 | Yes |
| 195 | TRIB2 |  |  | 636 | 0.158 | 0.7538 | Yes |
| 196 | E2F8 |  |  | 638 | 0.158 | 0.7556 | Yes |
| 197 | EXO1 |  |  | 643 | 0.157 | 0.7570 | Yes |
| 198 | ING2 |  |  | 646 | 0.157 | 0.7586 | Yes |
| 199 | SRRT |  |  | 657 | 0.155 | 0.7594 | Yes |
| 200 | PRIM1 |  |  | 660 | 0.155 | 0.7610 | Yes |
| 201 | CTCF |  |  | 691 | 0.151 | 0.7595 | Yes |
| 202 | LSM6 |  |  | 697 | 0.151 | 0.7607 | Yes |
| 203 | NEIL3 |  |  | 700 | 0.150 | 0.7623 | Yes |
| 204 | WDR76 |  |  | 702 | 0.150 | 0.7640 | Yes |
| 205 | ZNF22 |  |  | 709 | 0.149 | 0.7651 | Yes |
| 206 | RAD54L |  |  | 729 | 0.146 | 0.7647 | Yes |
| 207 | ZNF85 |  |  | 741 | 0.145 | 0.7652 | Yes |
| 208 | KBTBD2 |  |  | 742 | 0.145 | 0.7669 | Yes |
| 209 | FANCA |  |  | 743 | 0.145 | 0.7686 | Yes |
| 210 | POLA1 |  |  | 752 | 0.143 | 0.7695 | Yes |
| 211 | SNRPA |  |  | 754 | 0.143 | 0.7711 | Yes |
| 212 | FANCB |  |  | 770 | 0.142 | 0.7711 | Yes |
| 213 | SEPHS1 |  |  | 771 | 0.142 | 0.7728 | Yes |
| 214 | MNS1 |  |  | 774 | 0.142 | 0.7742 | Yes |
| 215 | NSMCE4A |  |  | 780 | 0.141 | 0.7753 | Yes |
| 216 | SKA3 |  |  | 790 | 0.139 | 0.7760 | Yes |
| 217 | CEP135 |  |  | 795 | 0.138 | 0.7772 | Yes |
| 218 | SASS6 |  |  | 811 | 0.137 | 0.7772 | Yes |
| 219 | MRTO4 |  |  | 864 | 0.131 | 0.7730 | Yes |
| 220 | ZNF519 |  |  | 868 | 0.131 | 0.7742 | Yes |
| 221 | KIF18B |  |  | 879 | 0.130 | 0.7746 | Yes |
| 222 | VEZF1 |  |  | 882 | 0.130 | 0.7759 | Yes |
| 223 | CCNF |  |  | 895 | 0.128 | 0.7761 | Yes |
| 224 | NIF3L1 |  |  | 922 | 0.126 | 0.7747 | Yes |
| 225 | RAB8A |  |  | 937 | 0.125 | 0.7747 | Yes |
| 226 | POLQ |  |  | 939 | 0.125 | 0.7761 | Yes |
| 227 | PTBP1 |  |  | 943 | 0.124 | 0.7772 | Yes |
| 228 | RP9 |  |  | 952 | 0.123 | 0.7778 | Yes |
| 229 | PLK4 |  |  | 986 | 0.121 | 0.7756 | Yes |
| 230 | C18orf54 |  |  | 1003 | 0.120 | 0.7752 | Yes |
| 231 | ILF3 |  |  | 1004 | 0.120 | 0.7766 | Yes |
| 232 | NR2C2AP |  |  | 1005 | 0.120 | 0.7780 | Yes |
| 233 | ILKAP |  |  | 1013 | 0.119 | 0.7787 | Yes |
| 234 | RCC1 |  |  | 1021 | 0.118 | 0.7793 | Yes |
| 235 | CEP152 |  |  | 1047 | 0.116 | 0.7779 | No |
| 236 | PNRC2 |  |  | 1049 | 0.115 | 0.7792 | No |
| 237 | CCDC138 |  |  | 1081 | 0.112 | 0.7771 | No |
| 238 | ELAVL1 |  |  | 1122 | 0.110 | 0.7739 | No |
| 239 | SUV39H2 |  |  | 1128 | 0.110 | 0.7747 | No |
| 240 | NUP205 |  |  | 1147 | 0.108 | 0.7740 | No |
| 241 | RBMX |  |  | 1186 | 0.105 | 0.7710 | No |
| 242 | C19orf57 |  |  | 1190 | 0.105 | 0.7719 | No |
| 243 | CENPJ |  |  | 1192 | 0.105 | 0.7730 | No |
| 244 | PKNOX1 |  |  | 1193 | 0.105 | 0.7743 | No |
| 245 | WDR62 |  |  | 1231 | 0.103 | 0.7714 | No |
| 246 | SAFB |  |  | 1233 | 0.102 | 0.7725 | No |
| 247 | CHEK2 |  |  | 1254 | 0.101 | 0.7715 | No |
| 248 | C5orf34 |  |  | 1282 | 0.099 | 0.7697 | No |
| 249 | ACBD6 |  |  | 1303 | 0.098 | 0.7686 | No |
| 250 | DDX11 |  |  | 1310 | 0.097 | 0.7691 | No |
| 251 | TRAIP |  |  | 1314 | 0.097 | 0.7699 | No |
| 252 | ZNF90 |  |  | 1320 | 0.097 | 0.7705 | No |
| 253 | KLHL23 |  |  | 1342 | 0.095 | 0.7693 | No |
| 254 | ARC |  |  | 1354 | 0.095 | 0.7692 | No |
| 255 | POLE |  |  | 1368 | 0.094 | 0.7689 | No |
| 256 | HNRNPU |  |  | 1369 | 0.094 | 0.7700 | No |
| 257 | BUD13 |  |  | 1408 | 0.092 | 0.7669 | No |
| 258 | THAP10 |  |  | 1424 | 0.091 | 0.7663 | No |
| 259 | RECQL4 |  |  | 1427 | 0.091 | 0.7672 | No |
| 260 | PDE6D |  |  | 1435 | 0.090 | 0.7675 | No |
| 261 | LIN54 |  |  | 1440 | 0.090 | 0.7681 | No |
| 262 | LIN9 |  |  | 1442 | 0.090 | 0.7690 | No |
| 263 | POLD1 |  |  | 1452 | 0.089 | 0.7691 | No |
| 264 | AAAS |  |  | 1453 | 0.089 | 0.7702 | No |
| 265 | ZNF704 |  |  | 1459 | 0.089 | 0.7707 | No |
| 266 | NAB1 |  |  | 1476 | 0.088 | 0.7699 | No |
| 267 | FIGNL1 |  |  | 1492 | 0.087 | 0.7693 | No |
| 268 | CHAF1B |  |  | 1530 | 0.085 | 0.7662 | No |
| 269 | EME1 |  |  | 1534 | 0.085 | 0.7669 | No |
| 270 | ZNF92 |  |  | 1546 | 0.084 | 0.7667 | No |
| 271 | CBX2 |  |  | 1560 | 0.084 | 0.7662 | No |
| 272 | DHX15 |  |  | 1565 | 0.083 | 0.7668 | No |
| 273 | RBL1 |  |  | 1569 | 0.083 | 0.7674 | No |
| 274 | KDM1A |  |  | 1577 | 0.082 | 0.7676 | No |
| 275 | RNASEH2A |  |  | 1579 | 0.082 | 0.7685 | No |
| 276 | CEP72 |  |  | 1625 | 0.081 | 0.7644 | No |
| 277 | GINS3 |  |  | 1639 | 0.080 | 0.7640 | No |
| 278 | ZNF93 |  |  | 1649 | 0.079 | 0.7639 | No |
| 279 | CDCA7 |  |  | 1684 | 0.078 | 0.7610 | No |
| 280 | RFWD3 |  |  | 1707 | 0.077 | 0.7595 | No |
| 281 | TP73 |  |  | 1719 | 0.076 | 0.7592 | No |
| 282 | NTHL1 |  |  | 1730 | 0.076 | 0.7590 | No |
| 283 | GEN1 |  |  | 1746 | 0.075 | 0.7582 | No |
| 284 | ING3 |  |  | 1778 | 0.073 | 0.7556 | No |
| 285 | STIL |  |  | 1784 | 0.073 | 0.7559 | No |
| 286 | DHX9 |  |  | 1786 | 0.073 | 0.7567 | No |
| 287 | FAM76B |  |  | 1800 | 0.073 | 0.7561 | No |
| 288 | HAUS3 |  |  | 1804 | 0.072 | 0.7567 | No |
| 289 | CDC25C |  |  | 1820 | 0.072 | 0.7558 | No |
| 290 | INCENP |  |  | 1833 | 0.071 | 0.7553 | No |
| 291 | C2orf80 |  |  | 1862 | 0.070 | 0.7531 | No |
| 292 | GABPB1 |  |  | 1894 | 0.069 | 0.7504 | No |
| 293 | PPP5C |  |  | 1905 | 0.068 | 0.7501 | No |
| 294 | SOX11 |  |  | 1922 | 0.067 | 0.7492 | No |
| 295 | SPATA5 |  |  | 1949 | 0.067 | 0.7471 | No |
| 296 | ZNF273 |  |  | 1956 | 0.066 | 0.7472 | No |
| 297 | CENPI |  |  | 1990 | 0.065 | 0.7443 | No |
| 298 | TUBA1A |  |  | 2004 | 0.064 | 0.7436 | No |
| 299 | ZNF232 |  |  | 2042 | 0.063 | 0.7402 | No |
| 300 | C1orf112 |  |  | 2044 | 0.063 | 0.7409 | No |
| 301 | DNA2 |  |  | 2070 | 0.062 | 0.7388 | No |
| 302 | TOP2B |  |  | 2082 | 0.062 | 0.7383 | No |
| 303 | GNB1 |  |  | 2089 | 0.061 | 0.7384 | No |
| 304 | PLEKHO1 |  |  | 2095 | 0.061 | 0.7386 | No |
| 305 | EXOSC2 |  |  | 2108 | 0.061 | 0.7380 | No |
| 306 | TOE1 |  |  | 2118 | 0.061 | 0.7377 | No |
| 307 | HNRNPL |  |  | 2136 | 0.060 | 0.7365 | No |
| 308 | NCOA5 |  |  | 2146 | 0.059 | 0.7362 | No |
| 309 | OIP5 |  |  | 2175 | 0.058 | 0.7338 | No |
| 310 | E2F2 |  |  | 2176 | 0.058 | 0.7345 | No |
| 311 | ZNF100 |  |  | 2215 | 0.057 | 0.7309 | No |
| 312 | SENP1 |  |  | 2218 | 0.057 | 0.7314 | No |
| 313 | KIF24 |  |  | 2258 | 0.055 | 0.7277 | No |
| 314 | MAP2 |  |  | 2271 | 0.055 | 0.7270 | No |
| 315 | CTDSPL2 |  |  | 2277 | 0.055 | 0.7271 | No |
| 316 | HCFC1 |  |  | 2319 | 0.053 | 0.7232 | No |
| 317 | ZNF530 |  |  | 2321 | 0.053 | 0.7237 | No |
| 318 | PHF2 |  |  | 2322 | 0.053 | 0.7243 | No |
| 319 | ZNF829 |  |  | 2333 | 0.053 | 0.7239 | No |
| 320 | ZNF593 |  |  | 2367 | 0.052 | 0.7208 | No |
| 321 | RBM14 |  |  | 2369 | 0.052 | 0.7213 | No |
| 322 | ZIK1 |  |  | 2404 | 0.051 | 0.7181 | No |
| 323 | CPSF4 |  |  | 2406 | 0.051 | 0.7186 | No |
| 324 | ZNF486 |  |  | 2410 | 0.051 | 0.7189 | No |
| 325 | KNTC1 |  |  | 2440 | 0.050 | 0.7163 | No |
| 326 | ZRANB3 |  |  | 2450 | 0.049 | 0.7158 | No |
| 327 | FANCC |  |  | 2472 | 0.049 | 0.7141 | No |
| 328 | CUL1 |  |  | 2498 | 0.048 | 0.7119 | No |
| 329 | ZNF43 |  |  | 2518 | 0.047 | 0.7103 | No |
| 330 | RAD54B |  |  | 2537 | 0.046 | 0.7089 | No |
| 331 | ARL5A |  |  | 2550 | 0.046 | 0.7081 | No |
| 332 | CDK14 |  |  | 2577 | 0.045 | 0.7057 | No |
| 333 | LCORL |  |  | 2583 | 0.045 | 0.7057 | No |
| 334 | SUV39H1 |  |  | 2604 | 0.044 | 0.7040 | No |
| 335 | NAB2 |  |  | 2634 | 0.043 | 0.7013 | No |
| 336 | PDIK1L |  |  | 2659 | 0.043 | 0.6991 | No |
| 337 | CCDC150 |  |  | 2692 | 0.042 | 0.6961 | No |
| 338 | NVL |  |  | 2715 | 0.041 | 0.6941 | No |
| 339 | ERI2 |  |  | 2721 | 0.041 | 0.6941 | No |
| 340 | PAXIP1 |  |  | 2729 | 0.041 | 0.6938 | No |
| 341 | RBBP4 |  |  | 2756 | 0.040 | 0.6914 | No |
| 342 | DCLRE1A |  |  | 2760 | 0.040 | 0.6915 | No |
| 343 | DUSP12 |  |  | 2765 | 0.040 | 0.6915 | No |
| 344 | CASP2 |  |  | 2794 | 0.039 | 0.6889 | No |
| 345 | DUSP28 |  |  | 2809 | 0.039 | 0.6878 | No |
| 346 | EPB41 |  |  | 2818 | 0.038 | 0.6874 | No |
| 347 | ESPL1 |  |  | 2838 | 0.038 | 0.6857 | No |
| 348 | ZNF48 |  |  | 2844 | 0.038 | 0.6856 | No |
| 349 | SP4 |  |  | 2864 | 0.037 | 0.6839 | No |
| 350 | MRPS17 |  |  | 3019 | 0.033 | 0.6672 | No |
| 351 | USP28 |  |  | 3025 | 0.033 | 0.6670 | No |
| 352 | MBNL3 |  |  | 3033 | 0.033 | 0.6666 | No |
| 353 | NFATC3 |  |  | 3073 | 0.032 | 0.6627 | No |
| 354 | ZNF738 |  |  | 3075 | 0.032 | 0.6630 | No |
| 355 | CDR2 |  |  | 3139 | 0.031 | 0.6563 | No |
| 356 | TNFAIP8L1 |  |  | 3154 | 0.031 | 0.6551 | No |
| 357 | LRRC40 |  |  | 3211 | 0.029 | 0.6492 | No |
| 358 | MTBP |  |  | 3311 | 0.027 | 0.6386 | No |
| 359 | KIAA1841 |  |  | 3346 | 0.027 | 0.6351 | No |
| 360 | ADNP2 |  |  | 3354 | 0.027 | 0.6346 | No |
| 361 | KDM2B |  |  | 3370 | 0.026 | 0.6333 | No |
| 362 | ATG4D |  |  | 3382 | 0.026 | 0.6324 | No |
| 363 | NAA16 |  |  | 3400 | 0.026 | 0.6308 | No |
| 364 | MIER3 |  |  | 3411 | 0.026 | 0.6300 | No |
| 365 | PHC1 |  |  | 3417 | 0.025 | 0.6297 | No |
| 366 | CUEDC1 |  |  | 3460 | 0.025 | 0.6254 | No |
| 367 | WIZ |  |  | 3468 | 0.024 | 0.6249 | No |
| 368 | RFX7 |  |  | 3469 | 0.024 | 0.6252 | No |
| 369 | ADD2 |  |  | 3551 | 0.023 | 0.6164 | No |
| 370 | ITPKA |  |  | 3553 | 0.023 | 0.6166 | No |
| 371 | ATRIP |  |  | 3570 | 0.022 | 0.6151 | No |
| 372 | FMNL3 |  |  | 3587 | 0.022 | 0.6136 | No |
| 373 | TMEM39B |  |  | 3630 | 0.022 | 0.6091 | No |
| 374 | HNRNPH1 |  |  | 3633 | 0.022 | 0.6092 | No |
| 375 | XPA |  |  | 3690 | 0.021 | 0.6032 | No |
| 376 | ZNF107 |  |  | 3691 | 0.021 | 0.6034 | No |
| 377 | KLHL22 |  |  | 3709 | 0.020 | 0.6018 | No |
| 378 | PDE7A |  |  | 3749 | 0.020 | 0.5977 | No |
| 379 | BCOR |  |  | 3806 | 0.019 | 0.5917 | No |
| 380 | SCLY |  |  | 3864 | 0.018 | 0.5856 | No |
| 381 | ZNF101 |  |  | 3874 | 0.018 | 0.5848 | No |
| 382 | ZNF689 |  |  | 3902 | 0.017 | 0.5820 | No |
| 383 | DCUN1D4 |  |  | 3951 | 0.016 | 0.5768 | No |
| 384 | NRF1 |  |  | 3952 | 0.016 | 0.5770 | No |
| 385 | FAM98B |  |  | 3955 | 0.016 | 0.5770 | No |
| 386 | SMARCAD1 |  |  | 3964 | 0.016 | 0.5763 | No |
| 387 | E2F6 |  |  | 3982 | 0.016 | 0.5746 | No |
| 388 | FNIP2 |  |  | 4006 | 0.015 | 0.5722 | No |
| 389 | CDKN1B |  |  | 4019 | 0.015 | 0.5711 | No |
| 390 | CCDC88A |  |  | 4035 | 0.015 | 0.5696 | No |
| 391 | SALL3 |  |  | 4113 | 0.014 | 0.5612 | No |
| 392 | GALE |  |  | 4116 | 0.014 | 0.5611 | No |
| 393 | LRRC28 |  |  | 4176 | 0.013 | 0.5547 | No |
| 394 | DBF4B |  |  | 4183 | 0.012 | 0.5542 | No |
| 395 | DUS3L |  |  | 4368 | 0.009 | 0.5338 | No |
| 396 | SETMAR |  |  | 4558 | 0.006 | 0.5129 | No |
| 397 | ZSCAN21 |  |  | 4677 | 0.004 | 0.4998 | No |
| 398 | FCHSD2 |  |  | 4812 | 0.003 | 0.4849 | No |
| 399 | CYTH2 |  |  | 4839 | 0.002 | 0.4821 | No |
| 400 | C7orf26 |  |  | 4870 | 0.002 | 0.4788 | No |
| 401 | FBXO17 |  |  | 4874 | 0.001 | 0.4784 | No |
| 402 | ZNF664 |  |  | 5008 | -0.000 | 0.4637 | No |
| 403 | PTCD1 |  |  | 5043 | -0.001 | 0.4599 | No |
| 404 | GPSM2 |  |  | 5127 | -0.002 | 0.4507 | No |
| 405 | ZNF786 |  |  | 5212 | -0.004 | 0.4414 | No |
| 406 | ZNF124 |  |  | 5373 | -0.006 | 0.4237 | No |
| 407 | PLEKHG2 |  |  | 5383 | -0.006 | 0.4227 | No |
| 408 | SECISBP2 |  |  | 5398 | -0.007 | 0.4213 | No |
| 409 | LRRC37B |  |  | 5448 | -0.008 | 0.4159 | No |
| 410 | PHACTR4 |  |  | 5483 | -0.008 | 0.4122 | No |
| 411 | ZNF678 |  |  | 5552 | -0.009 | 0.4048 | No |
| 412 | RNF169 |  |  | 5665 | -0.011 | 0.3924 | No |
| 413 | POLR1A |  |  | 5720 | -0.012 | 0.3866 | No |
| 414 | GAB1 |  |  | 5722 | -0.012 | 0.3866 | No |
| 415 | DOT1L |  |  | 5724 | -0.012 | 0.3866 | No |
| 416 | TET3 |  |  | 5739 | -0.012 | 0.3852 | No |
| 417 | MTG1 |  |  | 5858 | -0.014 | 0.3723 | No |
| 418 | PIF1 |  |  | 5869 | -0.014 | 0.3713 | No |
| 419 | CREBBP |  |  | 5967 | -0.016 | 0.3607 | No |
| 420 | TMSB15B |  |  | 5992 | -0.017 | 0.3583 | No |
| 421 | ICA1L |  |  | 6013 | -0.017 | 0.3562 | No |
| 422 | GTF2IRD1 |  |  | 6114 | -0.018 | 0.3453 | No |
| 423 | ZNF570 |  |  | 6177 | -0.020 | 0.3387 | No |
| 424 | IDH1 |  |  | 6208 | -0.020 | 0.3356 | No |
| 425 | FUS |  |  | 6216 | -0.020 | 0.3350 | No |
| 426 | TIA1 |  |  | 6487 | -0.025 | 0.3053 | No |
| 427 | PLEKHA8 |  |  | 6600 | -0.027 | 0.2932 | No |
| 428 | RBM4B |  |  | 6624 | -0.028 | 0.2909 | No |
| 429 | RSBN1L |  |  | 6643 | -0.028 | 0.2893 | No |
| 430 | MEX3A |  |  | 6667 | -0.029 | 0.2871 | No |
| 431 | DCLRE1C |  |  | 6712 | -0.030 | 0.2825 | No |
| 432 | ZNF669 |  |  | 6756 | -0.031 | 0.2781 | No |
| 433 | LBR |  |  | 7064 | -0.038 | 0.2444 | No |
| 434 | CRTC3 |  |  | 7354 | -0.046 | 0.2128 | No |
| 435 | TRRAP |  |  | 7402 | -0.047 | 0.2082 | No |
| 436 | TET2 |  |  | 7571 | -0.052 | 0.1901 | No |
| 437 | ATP9B |  |  | 7608 | -0.054 | 0.1867 | No |
| 438 | SPEN |  |  | 8024 | -0.068 | 0.1414 | No |
| 439 | AP4M1 |  |  | 8225 | -0.078 | 0.1201 | No |
| 440 | DGCR8 |  |  | 8245 | -0.079 | 0.1189 | No |
| 441 | CREB5 |  |  | 8279 | -0.082 | 0.1162 | No |
| 442 | ZNF117 |  |  | 8351 | -0.086 | 0.1093 | No |
| 443 | GTF2IRD2 |  |  | 8512 | -0.097 | 0.0927 | No |
| 444 | ZNF431 |  |  | 8628 | -0.105 | 0.0812 | No |
| 445 | TRA2A |  |  | 8773 | -0.119 | 0.0666 | No |
| 446 | ZNF75A |  |  | 8980 | -0.146 | 0.0454 | No |
| 447 | NTRK3 |  |  | 8986 | -0.146 | 0.0466 | No |
| 448 | MAML2 |  |  | 9028 | -0.154 | 0.0438 | No |
| 449 | PGAP1 |  |  | 9038 | -0.156 | 0.0447 | No |
Table: GSEA details [plain text format]

  

Fig 2: GAROFANO\_IAVARONE\_PPR\_SUBTYPE: Random ES distribution      
 Gene set null distribution of ES for **GAROFANO\_IAVARONE\_PPR\_SUBTYPE**
